# Smokers with Self-Reported Mental Health Conditions: A Case for Screening in the Context of Tobacco Cessation Services

**DOI:** 10.1371/journal.pone.0159127

**Published:** 2016-07-08

**Authors:** Gary J. Tedeschi, Sharon E. Cummins, Christopher M. Anderson, Robert M. Anthenelli, Yue-Lin Zhuang, Shu-Hong Zhu

**Affiliations:** 1 Moores Cancer Center, University of California San Diego, La Jolla, California, United States of America; 2 Department of Family Medicine and Public Health, University of California San Diego, La Jolla, California, United States of America; 3 Department of Psychiatry, University of California San Diego, La Jolla, California, United States of America; Centre for Addiction and Mental Health, CANADA

## Abstract

**Background:**

People with mental health conditions (MHC) smoke at high rates and many die prematurely from smoking-related illnesses. Smoking cessation programs, however, generally do not screen for MHC. This study examined the utility of MHC screening in a large tobacco quitline to determine whether self-reported MHC predicts service utilization and quitting behaviors.

**Methods & Findings:**

A brief set of question on MHC was embedded in the routine intake of a state quitline, and 125,261 smokers calling from June 2012 to September 2015 were asked the questions. Quit attempt rate and 6-month success rate were analyzed for a random subset of participants. Overall, 52.2% of smokers reported at least one MHC. Demographic patterns like gender or ethnic difference in self-reported MHC were similar to that in the general population. Depression disorder was reported most often (38.6%), followed by anxiety disorder (33.8%), bipolar disorder (17.0%), drug/alcohol abuse (11.9%), and schizophrenia (7.9%). Among those reporting any MHC, about two-thirds reported more than 1 MHC. Smokers with MHC received more counseling than smokers with no MHC. Quit attempt rates were high for all three groups (>70%). The probability of relapse was greater for those with more than one MHC than for those with one MHC (p<0.005), which in turn was greater than those with no MHC (p < .01). The six-month prolonged abstinence rates for the three conditions were, 21.8%, 28.6%, and 33.7%, respectively. The main limitation of this study is the use of a non-validated self-report question to assess MHC, even though it appears to be useful for predicting quitting behavior.

**Conclusions:**

Smokers with MHC actively seek treatment to quit. Smoking cessation services can use a brief set of questions to screen for MHC to help identify smokers in need of more intensive treatment to quit smoking.

## Introduction

Smoking prevalence is higher among those with mental health conditions (MHC) than among those without [1,2]. Smokers with MHC have a harder time quitting than smokers without MHC [3], and experience tobacco-related illnesses at a greater rate [4–8]. There has been a call for clinicians and researchers to focus more on tobacco cessation for people with MHC [9–14].

Tobacco cessation services, however, generally do not screen for MHC [12,15]. Screening for MHC can be time consuming, and many cessation service providers do not feel they are equipped to treat MHC [16–18]. On the other hand, mental health services tend to view tobacco dependence as a low priority compared with other treatment concerns [19–21]. The lack of screening for MHC in tobacco cessation services and low attention to tobacco dependence from mental health programs mean there are missed opportunities to help smokers with MHC in treatment settings [12].

This study presents a case for screening for MHC in tobacco cessation services, specifically in the context of a state quitline. Quitlines are telephone-based counseling services for smoking cessation [22]. They are major providers of tobacco cessation services in the U.S. The 51 state quitlines (including D.C.) collectively assist nearly 430,000 unique tobacco users annually [23]. There has been a growing interest in quitline utilization by smokers with MHC, and in what role quitlines can play in cessation efforts for these smokers [19,24–28].

The main objective of this study is to show that a brief set of questions is useful in identifying smokers with self-reported MHC and in predicting outcomes for cessation. The questions do not aim to diagnose specific mental health conditions. Instead, they are used to generate information that could aid in treatment planning and counseling to improve cessation rates for smokers with MHC.

To be practical for quitlines, a MHC assessment must be brief. Quitline callers typically expect the intake process to be short and focused. Using one or more diagnostic instruments to assess mental health, although possible [24,29], would significantly increase intake time and could be perceived by smokers as irrelevant and intrusive. This is especially true since quitlines do not treat MHC. This concern has prevented many cessation services from screening for MHC. On the other hand, a brief set of questions on self-reported mental health conditions is practical and can be helpful in devising appropriate cessation interventions.

To test this approach, we embedded a self-report measure for mental health in the intake protocol of the California Smokers’ Helpline, the longest running state quitline in the U.S. The present study examines the feasibility and usefulness of employing such a measure. Specifically, the value of the measure was tested by examining if answers to the MHC questions predicted treatment utilization and smoking cessation outcomes.

## Methods

### Participants

Study participants were adult smokers who called the California Smokers’ Helpline (CSH) between June 2012-September 2015. CSH is California’s quitline, a free statewide telephone-based tobacco cessation service. The Helpline provides services in English, Spanish, Mandarin, Cantonese, Vietnamese, and Korean. This study included only those who called the English and Spanish language lines. Since participants lived in different locations throughout California, and took part in the study by telephone, oral consent was obtained and recorded in the Helpline data base. All research activity for this study, including the consent procedure, was approved by the University of California, San Diego Human Research Protections Program (#080517).

### Procedure

#### Intake

Helpline staff used a computer-assisted telephone interview (CATI) system that presented the mental health questions embedded in the Helpline standard intake. The standard intake interview included items such as demographics, health insurance status, smoking status, tobacco consumption, and health conditions. The mental health questions followed questions on physical health.

#### Services

All counselors’ contact with participants was logged in the Helpline database, including call date, call attempts, and type and length of calls. The counseling protocol used during this study has been described elsewhere in detail [30] and has been shown to be effective in clinical trials [31,32]. Counseling was provided by trained counselors, and included a comprehensive initial session to prepare for a quit date and up to four follow-up calls at critical times, based on the probability of relapse [33].

#### Measures

Baseline measures to assess MHC at intake were consistent with those recommended by the North American Quitline Consortium (NAQC) as an optional addition to the minimal data set [34]. Helpline callers were asked “Do you have any mental health conditions such as an anxiety disorder? Depression disorder? Bipolar disorder? Schizophrenia? Alcohol/drug abuse?” Staff recorded a yes or no for each condition. When smokers stated they did not know (about 1% of the time), staff followed with, “Has a doctor or other health care professional told you that you have a mental health condition?” If smokers responded yes, they were asked to specify which conditions, and staff coded responses accordingly.

The evaluation measure used to determine engagement in services was number of counseling sessions received. Quitting was assessed by two measures: quit attempts and prolonged abstinence. A quit attempt was defined as quitting for at least 24 hours. For those that made a quit attempt, prolonged abstinence was defined as not smoking for at least 180 days at the time of evaluation. If a smoker made more than one quit attempt, the first quit attempt was used in the analysis of prolonged abstinence.

#### Evaluation

Seven-month post enrollment evaluation interviews were conducted by telephone by research assistants, independent of the counselors. Based on standard Helpline evaluation procedures, with oversampling of pregnant smokers and households with children under age five, an average of 9% of participants were randomly selected for evaluation. Evaluations were conducted from January 2013-February 2015.

#### Statistical analysis

To examine the effect of number of MHC on treatment engagement, all participants were categorized into three groups: those with no MHC, one MHC, and more than one MHC. Confidence intervals (95%) were calculated around parameters and chi-squares were used to evaluate differences in proportions. Survival analysis was performed by using Kaplan-Meier method to generate relapse curves for the three MHC groups. Log-rank tests with Sidak multiple-comparison adjustment were used to compare the relapse curves among the three MHC groups. All statistical analyses were conducted using SAS statistical package, version 9.4 [35].

## Results

A total of 125,261 adult smokers who called the quitline were asked the mental health questions during the study period. Of these, 97.8% answered the question on anxiety, 98.0% on depression, 97.4% on bipolar, 98.4% schizophrenia, and 98.9% on drug/alcohol abuse.

Overall, 52.2% of smokers reported at least one MHC, as shown in Table 1. Depression was the most frequently reported condition (38.4%), followed by anxiety disorder (35.1%), bipolar disorder (17.1%), drug/alcohol abuse (11.8%), and schizophrenia (8.1%).

**Table 1:** Self-reported mental health condition by demographic characteristics.

Characteristic	Anxiety	Depression	Bipolar	Schizophrenia	Drug/Alcohol	Any	Any
N = 125,261	% Yes	% Yes	% Yes	% Yes	% Yes	% Yes	95% CI
**All**	35.1	38.4	17.1	8.1	11.8	52.2	52.0–52.5
**Gender**							
Male	26.9	30.5	13.9	9.4	14.7	45.7	45.2–46.1
Female	41.7	44.8	19.6	7.1	9.6	57.5	57.1–57.9
**Age**							
<18	36.2	39.1	11.8	5.9	13.0	46.2	34.0–58.3
18–24	31.5	31.2	17.3	6.0	15.0	48.9	47.8–50.1
25–44	35.3	35.4	18.4	8.7	14.4	51.8	51.3–52.2
45–64	36.7	42.2	17.5	8.7	10.7	54.5	54.1–54.9
65+	26.4	32.4	8.0	3.4	5.7	41.8	40.8–42.9
**Education**							
< = 12	34.8	39.1	17.9	10.0	12.8	53.1	52.7–53.5
>12	35.5	37.7	16.1	5.9	10.7	51.3	50.9–51.7
**Race**							
White	38.7	40.3	18.4	6.8	12.2	55.3	54.9–55.7
Black	27.2	35.3	15.9	10.5	9.9	46.2	45.5–46.8
Hispanic	31.2	35.2	12.7	7.8	12.7	48.9	48.1–49.6
Asian/PI	23.4	26.2	11.4	8.5	8.7	37.5	35.7–39.2
American Indian	42.7	44.7	21.7	9.5	13.9	60.3	58.2–62.5
Multi-racial	43.8	44.9	22.2	10.7	14.3	61.1	60.0–62.1
Others	28.5	33.7	12.6	6.2	9.2	42.1	39.1–45.1

Table 1 also shows demographic characteristics of smokers by MHC. Overall, women had a significantly higher MHC rate than men did (57.5% vs. 45.7%). However, this varied by condition. Women were more likely to report anxiety, depression and bipolar disorder than men. But they were less likely to report schizophrenia or drug/alcohol problems (all gender differences by condition are statistically significant, p < .001).

The self-reported MHC rate increased with age, except for those aged 65 or older. Those participants had a MHC rate that was similar to the youngest age group. Those with less education had a slightly higher MHC rate than those with more education.

Differences by race/ethnicity were noticeable. Overall, multiracial participants and American Indians had the highest MHC rates, at 61.1% and 60.3% respectively. They were followed by Whites (55.3%), Hispanics (48.9%), Blacks (46.2%) and Asians/Pacific Islanders (37.5%). The category “others” in Table 1 includes all those who could not be categorized.

Table 2 shows the overlap in reported conditions. Each column provides the percentage of reported conditions listed in the row and conditions listed in the column. For example, column 1 shows that 77.8% of those who reported anxiety disorder also reported depression. Column 1 also shows that of those who reported anxiety disorder, 36.2% also reported bipolar disorder. Overlap between categories was considerable and included a combination of conditions not typically diagnosed concurrently, like bipolar disorder and schizophrenia. Depression was the condition most likely to co-occur with another condition. This was expected given that depression was the most frequently reported condition.

**Table 2:** Overlap in reported mental health condition.

Characteristic	Anxiety	Depression	Bipolar	Schizophrenia	Drug/Alcohol
	%	%	%	%	%
	N = 43,017	N = 47,193	N = 20,847	N = 10,040	N = 14,679
**Anxiety**	100	71.4	73.1	68.6	49.3
**Depression**	77.8	100	79.2	73.0	54.6
**Bipolar**	36.2	36.0	100	54.8	28.6
**Schizophrenia**	16.0	15.6	26.2	100	13.9
**Drug/Alcohol**	16.6	16.8	19.6	20.2	100

Specific MHC aside, 18.5% of smokers reported one MHC, 17.6% reported two, 11.0% three, and 5.2% four or more. Thus, among those who reported having a MHC, about two thirds reported having more than one. To examine the effects of comorbidity, therefore, participants were categorized into three groups: those with no MHC (47.8%), one MHC (18.5%) and more than one MHC (33.7%).

Table 3 examines participants who received follow-up evaluation and shows the degree to which they engaged in treatment. Smokers with one MHC (76.1%) and more than one MHC (78.8%) were more likely to receive at least one counseling session than those with no MHC (69.9%), while differences for those with one MHC and more than one MHC were not statistically significant. The same pattern emerged for total number of counseling sessions received: smokers with one MHC or more than one MHC received more sessions than smokers with no MHC, but there is no significant difference between the two MHC groups. The use of pharmacotherapy appears to be less different across groups, although smokers with more than one MHC (68.1%) used cessation medication at a higher rate than those with no MHC (64.8%, p<0.05).

**Table 3:** Use of counseling and pharmacotherapy by mental health condition (MHC).

Characteristic	0 MHC %	1 MHC %	>1 MHC %	1 MHC vs. 0 MHC	>1 MHC vs. 0 MHC	>1 MHC vs. 1 MHC
	N = 2,070	N = 770	N = 1,320	P-value	P-value	P-value
**% received initial session**	69.9	76.1	78.8	<.005	<.001	.15
**Total sessions received ≥3**	38.3	44.5	47.7	<.005	<.001	.16
**Use of any pharmacotherapy**	64.8	65.2	68.1	.84	<.05	.17

**Fig 1:**
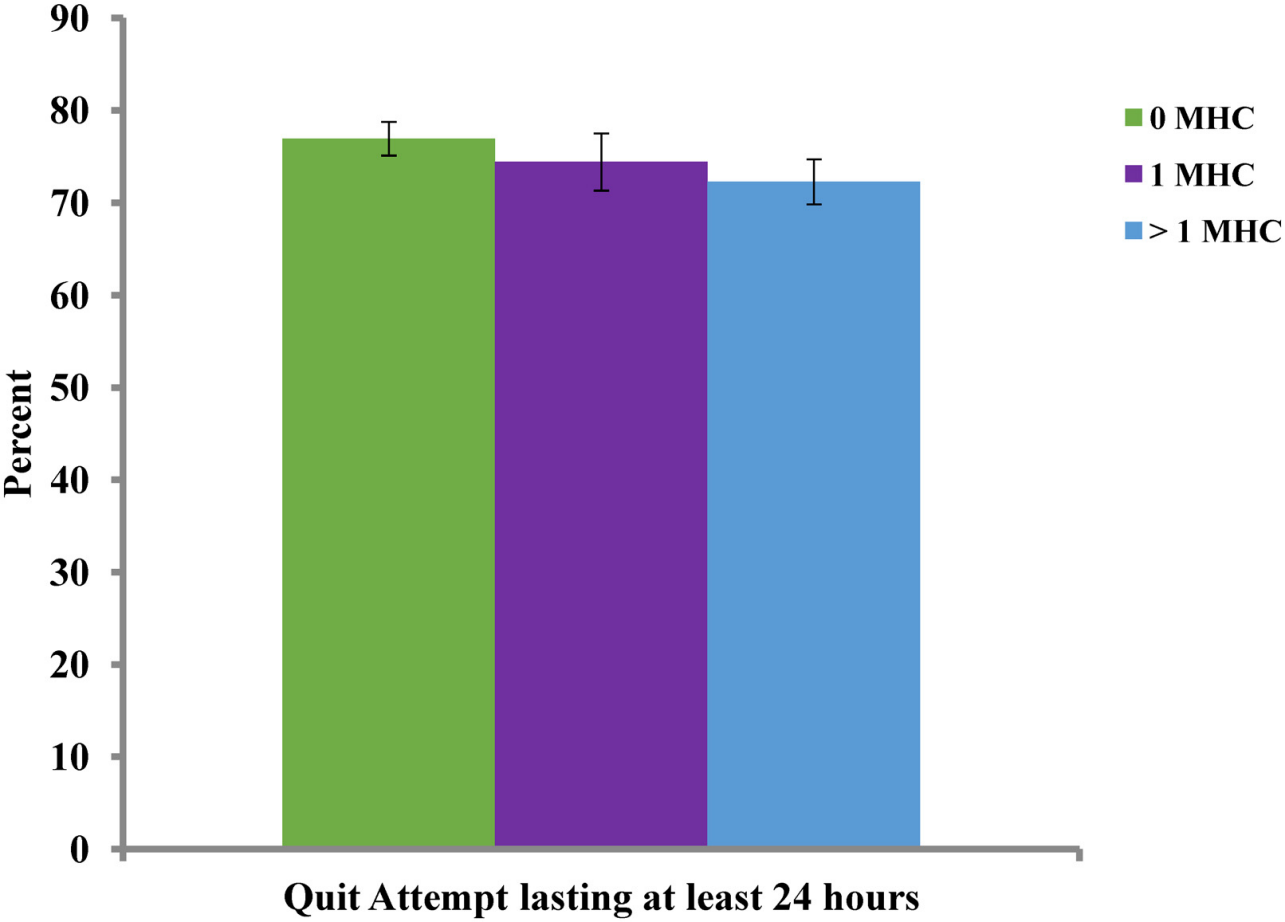
Quit attempts by mental health condition (MHC).

**Fig 2:**
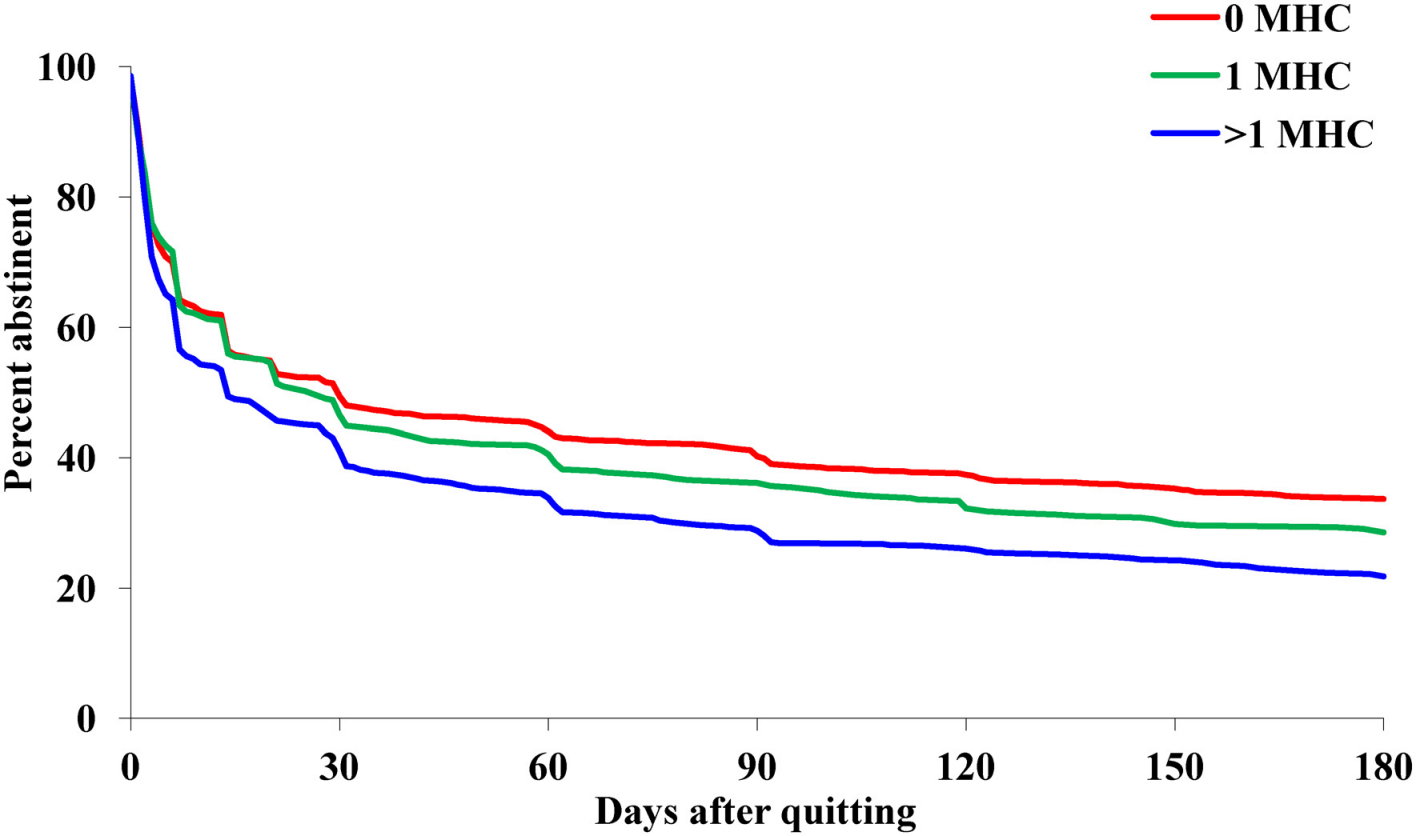
Relapse curves for those who made a quit attempt, by mental health condition (MHC).

Figs 1 and 2 show the results from those smokers who were randomly selected to be followed (N = 8,397) and were reached (N = 4,160, 49.54%) for evaluation seven months after enrollment. Fig 1 shows that the rate at which smokers made a quit attempt was high across all three groups. At least 70% of all three groups made a 24-hour quit attempt, although smokers with more than one MHC were less likely to make a quit attempt than those with no MHC (72.3% vs. 76.9%; p < .01). There was no statistically significant difference between those with one MHC and more than one MHC or between those with one MHC and no MHC.

Fig 2 presents the relapse curves for those who made a 24-hour quit attempt. A comparison between these curves shows that the probability of relapse was greater for those with more than one MHC than for those with one MHC (Log-rank χ^2^ = 11.52, p<0.005), which in turn was greater than those with no MHC (Log-rank χ^2^ = 8.32, p < .01). The six-month prolonged abstinence rates for the three conditions were, 21.8%, 28.6%, and 33.7%, respectively.

## Discussion

Results from this study of over 125,000 smokers showed that a simple self-report measure of MHC can be used as part of a routine quitline intake. This measure was informative, as item responses were associated with treatment utilization and smoking cessation rates. Three results stand out: First, a large proportion of smokers calling the statewide service reported at least one mental health condition (MHC). Second, those who reported MHC tended to use more counseling. Third, callers with or without MHC were all highly motivated to make a quit attempt, but those with MHC were more likely to relapse after a quit attempt.

Over half (52.2%) of adult smokers calling the Helpline reported at least one MHC. This study did not attempt to verify a clinical diagnosis for each self-reported MHC. It is expected that conditions such as depression and anxiety might be over-reported, yielding false positives when a simple screening measure is used [36]. On the other hand, it is less likely that smokers would report having bipolar disorder or schizophrenia, unless they had been diagnosed by a trained health professional. These terms can still be stigmatizing, especially among certain groups such as Asian Americans for example [37]. Thus, one might argue the tendency is to under-report rather than over-report these conditions. The same could be said about alcohol and drug abuse, which is often under-reported [38–41].

Over-reporting, however, can come in another form. The overlap in smokers’ self-report of bipolar disorder and schizophrenia is a good example. Bipolar disorder and schizophrenia, while having some shared characteristics, are not typically diagnosed concurrently. A more likely scenario is that smokers were diagnosed with one condition at one point and later diagnosed with the other, as shown in previous work [42,43]. As noted earlier, the mental health questions for this study were not intended to diagnose specific MHC, but rather to obtain a broad measure of psychological health. It is for this reason that in the analyses smokers were grouped by number of self-reported MHC, not by condition.

The results from an earlier study from the California Smokers’ Helpline [24] could provide an estimate of how much over-reporting might occur if simple screening questions are used to assess MHC. In the present study, 38.5% reported having depression. For the cited study, 24.2% of Helpline callers met criteria for Current Major Depressive Disorder (MDD) using the PHQ-9 [24]. Put another way, less than two thirds of those reporting depression might meet criteria for a diagnosis of MDD. If the same ratio was applied to the overall self-reported MHC in this study, then the proportion of smokers having at least one mental health condition among the Helpline callers, conservatively, would be about 33% rather than 52%, as shown in Table 1.

However, the overall demographic pattern among those who reported MHC in this study is similar to that of the general population [44,45]. For example, the higher rate of self-reported MHC for women is similar to that from the National Survey of Drug Use and Health [2]. The rank order across ethnicities is also similar, with American Indians and multiracial individuals ranking at the top, followed by Whites, Hispanics, Blacks and Asians. It appears that smokers calling the Helpline reflect the general population in their relative likelihood of reporting a MHC when compared by demographics. They differ, however, from the general population in their absolute rates of self-reported MHC, as rates in the present study were higher.

This large studysupports previous work that also examined self-reported MHC for smokers seeking treatment [26–28], but makes the following unique contribution. It shows that smokers’ self-report of MHC may be taken at face value and used to guide treatment. The study avoided the more time-consuming process of using formal diagnostic measures, as in one of our earlier studies [24]. The time needed to formally assess even one condition, such as Major Depression, would be burdensome for treatment-seeking smokers who have more immediate cessation questions. Therefore, the present study used a brief self-report measure and then grouped smokers by the number of MHC reported. The basic assumption was that while a smoker’s self-report might not be as accurate as a formal clinical diagnosis, it would reflect a self-assessment of the participant’s mental health challenges. For most counseling services that focus on nicotine dependence treatment, it is not expected that they also provide mental health treatment. However, some measure of mental health can be used to guide treatment plans, as it is likely that smokers with more mental health concerns need more intensive service [10,16,46]. We believe a short assessment such as the one used in this study can serve that purpose. It strikes a balance between time-consuming diagnostic procedures and not assessing MHC at all.

One piece of evidence supporting this approach is the counseling utilization data shown in Table 3. Smokers with more MHC received more counseling sessions, even though the protocol called for the same number of sessions for all smokers. Smokers with one MHC received more sessions than smokers with no MHC, and smokers with more than one MHC received more sessions than smokers with one MHC. It is conceivable that smokers with more mental health concerns were more receptive to follow-up appointments, possibly even requesting extra sessions. Additionally, counselors may have encouraged additional sessions for smokers with MHC based on their intuitive assessments of clinical need during counseling sessions. In other words, the dynamic interaction between smokers and counselors may be reflected in the number of sessions received, which turned out to be correlated with self-reported MHC.

It should be noted that even though the level of counseling service provided to the smokers increased with the number of MHC, the difference in relapse probability still went in the opposite direction (Fig 2). In other words, the additional help for those with more MHC was insufficient to counter the effect of MHC on relapse. The number of sessions added was limited because the protocol called for an equal number of sessions for all smokers; there was no formal guideline to provide smokers with MHC more sessions during the study period. In the end, smokers with more MHC were still more likely to relapse.

The results from this study suggest that smokers with MHC may need more intensive treatment in order to succeed in quitting at the same level as those with no MHC. This is especially true for those who have more than one MHC. A more formal test of intensive treatment for smokers with MHC is urgently needed because smokers with MHC are actively seeking treatment to quit smoking.

### Limitations

The main limitation of this study is the use of non-validated self-report questions to assess MHC. Brief screening questions for conditions such as depression and anxiety can result in false positives [36]. Smokers may have responded as if asked whether they *ever* had MHC, rather than *currently* had MHC as implied in the question. “*Do you have* any mental health conditions such as…” The intake protocol in this study did not require smokers to clarify when they received their MHC diagnoses, which could lead to higher than usual endorsement of multiple conditions.

## Conclusions

Smokers with MHC call quitlines in large numbers. They are motivated to quit, but less likely to succeed in quitting than smokers with no MHC. Thus, it is imperative to develop interventions to help them quit successfully. Telephone quitlines are one of the few behavioral services that reach smokers in large numbers and these programs have been disseminated internationally [22]. However, the efficacy of these quitlines for smokers with MHC is not well established. This is in part due to the fact that most cessation services do not measure MHC or do not use it to guide the treatment. This study shows that even a brief measure of MHC can predict the relapse probability of a given quit attempt. This suggests that smoking cessation services should routinely assess for MHC and use the information to guide treatment. One way to improve quit rates for smokers with MHC is to employ protocols that have more counseling sessions or longer use of cessation pharmacotherapy [10,16,46]. Another way, especially for those with more than one MHC, would be more intensive interventions along with referral to and collaboration with mental health clinicians for concurrent mental health treatment. A more formal test of whether a more intensive treatment can produce a higher quit rate for smokers with MHC is needed.

As a starting point, quitlines could use a general measure of psychiatric health at intake to identify smokers with MHC, such as the one used in this study. The fact that a high number of smokers with MHC call quitlines for assistance presents an excellent opportunity to increase tobacco dependency treatment utilization in people with MHC, thereby improving their quality of life. Quitline services are free, easy to access, and provided by well-trained staff. They can continue to play a role in helping smokers with MHC quit, and can be a resource for mental health clinicians working with smokers with MHC.

## Supporting Information

S1 Data Tables 1 and 2. (CSV)

S2 Data Table 3 and Fig 1. (CSV)

S3 Data Fig 2. (CSV)

S1 Text STROBE Statement. (DOCX)

S2 Text Data Dictionary. (DOCX)

## References

[1] Centers for Disease Control and Prevention (CDC). Vital signs: current cigarette smoking among adults aged ≥18 years with mental illness—United States, 2009–2011. MMWR Morb Mortal Wkly Rep. 2013;62: 81–87. 23388551 PMC4604817

[2] Substance Abuse and Mental Health Services Administration. Results from the 2012 National Survey on Drug Use and Health: mental health findings [Internet]. Rockville, MD: Substance Abuse and Mental Health Services Administration; 2013 Available: http://www.samhsa.gov/data/sites/default/files/NSDUHmhfr2012/NSDUHmhfr2012.pdf

[3] Trosclair A, Dube SR. Smoking among adults reporting lifetime depression, anxiety, anxiety with depression, and major depressive episode, United States, 2005–2006. Addict Behav. 2010;35: 438–443. 20079577 10.1016/j.addbeh.2009.12.011

[4] Olfson M, Gerhard T, Huang C, Crystal S, Stroup TS. Premature Mortality Among Adults With Schizophrenia in the United States. JAMA Psychiatry. 2015; 1–10. 10.1001/jamapsychiatry.2015.1737 26509694

[5] Druss BG, Zhao L, Von Esenwein S, Morrato EH, Marcus SC. Understanding excess mortality in persons with mental illness: 17-year follow up of a nationally representative US survey. Med Care. 2011;49: 599–604. 21577183 10.1097/MLR.0b013e31820bf86e

[6] Parks J, Svendsen D, Singer P, Foti ME, Mauer B. Morbidity and mortality in people with serious mental illness [Internet]. National Association of State Mental Health Program Directors (NASMHPD) Medical Directors Council Alexandria, VA; 2006 Available: http://theempowermentcenter.net/Articles/Technical%20Report%20on%20Morbidity%20and%20Mortaility%20-%20Final%2011-06.pdf

[7] Ziedonis DM, Williams JM. Management of smoking in people with psychiatric disorders: Curr Opin Psychiatry. 2003;16: 305–315.

[8] Campion J, Checinski K, Nurse J, McNeill A. Smoking by people with mental illness and benefits of smoke-free mental health services. Adv Psychiatr Treat. 2008;14: 217–228. 10.1192/apt.bp.108.005710

[9] Cerimele JM, Halperin AC, Saxon AJ. Tobacco use treatment in primary care patients with psychiatric illness. J Am Board Fam Med JABFM. 2014;27: 399–410. 10.3122/jabfm.2014.03.130252 24808119 PMC4269522

[10] Cook BL, Wayne GF, Kafali EN, Liu Z, Shu C, Flores M. Trends in smoking among adults with mental illness and association between mental health treatment and smoking cessation. JAMA. 2014;311: 172–182. 10.1001/jama.2013.284985 24399556 PMC5555156

[11] Lembke A, Humphreys K. A call to include people with mental illness and substance use disorders alongside “regular” smokers in smoking cessation research. Tob Control. 2015; 10.1136/tobaccocontrol-2014-052215 25882685

[12] Schroeder SA, Morris CD. Confronting a neglected epidemic: tobacco cessation for persons with mental illnesses and substance abuse problems. Annu Rev Public Health. 2010;31: 297–314 1p following 314. 10.1146/annurev.publhealth.012809.103701 20001818

[13] Williams JM, Steinberg ML, Griffiths KG, Cooperman N. Smokers with behavioral health comorbidity should be designated a tobacco use disparity group. Am J Public Health. 2013;103: 1549–1555. 10.2105/AJPH.2013.301232 23865661 PMC3776478

[14] Tidey JW, Miller ME. Smoking cessation and reduction in people with chronic mental illness. BMJ. 2015;351: h4065 10.1136/bmj.h4065 26391240 PMC4707528

[15] Phillips KM, Brandon TH. Do psychologists adhere to the clinical practice guidelines for tobacco cessation? A survey of practitioners. Prof Psychol Res Pract. 2004;35: 281–285. 10.1037/0735-7028.35.3.281

[16] Aubin H-J, Rollema H, Svensson TH, Winterer G. Smoking, quitting, and psychiatric disease: a review. Neurosci Biobehav Rev. 2012;36: 271–284. 10.1016/j.neubiorev.2011.06.007 21723317

[17] Grant BF, Hasin DS, Chou SP, Stinson FS, Dawson DA. Nicotine dependence and psychiatric disorders in the United States: results from the national epidemiologic survey on alcohol and related conditions. Arch Gen Psychiatry. 2004;61: 1107–1115. 10.1001/archpsyc.61.11.1107 15520358

[18] Siru R, Hulse GK, Tait RJ. Assessing motivation to quit smoking in people with mental illness: a review. Addict Abingdon Engl. 2009;104: 719–733. 10.1111/j.1360-0443.2009.02545.x 19413788

[19] Rogers E, Sherman S. Tobacco use screening and treatment by outpatient psychiatrists before and after release of the American Psychiatric Association treatment guidelines for nicotine dependence. Am J Public Health. 2014;104: 90–95. 10.2105/AJPH.2013.301584 24228666 PMC3910050

[20] Himelhoch S, Daumit G. To whom do psychiatrists offer smoking-cessation counseling? Am J Psychiatry. 2003;160: 2228–2230. 14638595 10.1176/appi.ajp.160.12.2228

[21] Thorndike AN, Stafford RS, Rigotti NA. US physicians’ treatment of smoking in outpatients with psychiatric diagnoses. Nicotine Tob Res Off J Soc Res Nicotine Tob. 2001;3: 85–91. 10.1080/14622200020032132 11260815

[22] Anderson CM, Zhu S-H. Tobacco quitlines: looking back and looking ahead. Tob Control. 2007;16: i81–i86. 10.1136/tc.2007.020701 18048638 PMC2598521

[23] Saul J, Davis R, North American Quitline Consortium. Results from the 2012 NAQC Annual Survey of Quitlines [Internet]. Jul 2013 [Accessed 8 Jun 2015]. Available: https://c.ymcdn.com/sites/www.naquitline.org/resource/resmgr/2012_annual_survey/oct23naqc_2012_final_report_.pdf

[24] Hebert KK, Cummins SE, Hernández S, Tedeschi GJ, Zhu S-H. Current major depression among smokers using a state quitline. Am J Prev Med. 2011;40: 47–53. 10.1016/j.amepre.2010.09.030 21146767 PMC3006168

[25] Morris CD, Tedeschi GJ, Waxmonsky JA, May M, Giese AA. Tobacco quitlines and persons with mental illnesses: perspective, practice, and direction. J Am Psychiatr Nurses Assoc. 2009;15: 32–40. 10.1177/1078390308330050 21665792

[26] Kerkvliet JL, Wey H, Fahrenwald NL. Cessation among state quitline participants with a mental health condition. Nicotine Tob Res Off J Soc Res Nicotine Tob. 2015;17: 735–741. 10.1093/ntr/ntu239 25385874

[27] Vickerman KA, Schauer GL, Malarcher AM, Zhang L, Mowery P, Nash CM. Quitline Use and Outcomes among Callers with and without Mental Health Conditions: A 7-Month Follow-Up Evaluation in Three States. BioMed Res Int. 2015;2015: 817298 10.1155/2015/817298 26273647 PMC4529922

[28] Lukowski AV, Morris CD, Young SE, Tinkelman D. Quitline Outcomes for Smokers in 6 States: Rates of Successful Quitting Vary by Mental Health Status. Nicotine Tob Res Off J Soc Res Nicotine Tob. 2015;17: 924–930. 10.1093/ntr/ntu252 26180216

[29] Pinto-Meza A, Serrano-Blanco A, Peñarrubia MT, Blanco E, Haro JM. Assessing depression in primary care with the PHQ-9: can it be carried out over the telephone? J Gen Intern Med. 2005;20: 738–742. 10.1111/j.1525-1497.2005.0144.x 16050884 PMC1490180

[30] Zhu S-H, Tedeschi GJ, Anderson CM, Pierce JP. Telephone counseling for smoking cessation: What’s in a call? J Couns Dev. 1996;75: 93–102. 10.1002/j.1556-6676.1996.tb02319.x

[31] Zhu S-H, Stretch V, Balabanis M, Rosbrook B, Sadler G, Pierce JP. Telephone counseling for smoking cessation: effects of single-session and multiple-session interventions. J Consult Clin Psychol. 1996;64: 202–211. 8907100 10.1037//0022-006x.64.1.202

[32] Zhu S-H, Anderson CM, Tedeschi GJ, Rosbrook B, Johnson CE, Byrd M, et al Evidence of real-world effectiveness of a telephone quitline for smokers. N Engl J Med. 2002;347: 1087–1093. 10.1056/NEJMsa020660 12362011

[33] Zhu S-H, Pierce JP. A new scheduling method for time-limited counseling. Prof Psychol Res Pract. 1995;26: 624–625. 10.1037/0735-7028.26.6.624

[34] North American Quitline Consortium. MDS Standard Optional Questions: Behavioral Health Screening [Internet]. 15 7 2011 [Accessed 24 May 2016]. Available: http://c.ymcdn.com/sites/www.naquitline.org/resource/resmgr/mdsta2010/mds_optional_bhaf_screening_.pdf

[35] SAS Institute Inc. SAS^®^ 9.4 Language Reference. 3rd ed Cary, NC: SAS Institute Inc; 2014.

[36] Mitchell AJ, Coyne JC. Do ultra-short screening instruments accurately detect depression in primary care? A pooled analysis and meta-analysis of 22 studies. Br J Gen Pract J R Coll Gen Pract. 2007;57: 144–151. PMC2034175 17263931

[37] Chu J, Sue S. Asian American Mental Health: What We Know and What We Don’t Know. Online Read Psychol Cult. 2011;3 10.9707/2307-0919.1026

[38] Bahorik AL, Newhill CE, Queen CC, Eack SM. Under-reporting of drug use among individuals with schizophrenia: prevalence and predictors. Psychol Med. 2014;44: 61–69. 10.1017/S0033291713000548 23551851

[39] Hormes JM, Gerhardstein KR, Griffin PT. Under-reporting of alcohol and substance use versus other psychiatric symptoms in individuals living with HIV. AIDS Care. 2012;24: 420–423. 10.1080/09540121.2011.608795 21942759 PMC3288469

[40] Morral AR, McCaffrey D, Iguchi MY. Hardcore drug users claim to be occasional users: drug use frequency underreporting. Drug Alcohol Depend. 2000;57: 193–202. 10.1016/S0376-8716(99)00048-4 10661670

[41] Stockwell T, Donath S, Cooper-Stanbury M, Chikritzhs T, Catalano P, Mateo C. Under-reporting of alcohol consumption in household surveys: a comparison of quantity–frequency, graduated–frequency and recent recall. Addiction. 2004;99: 1024–1033. 10.1111/j.1360-0443.2004.00815.x 15265099

[42] Bowden CL. Strategies to reduce misdiagnosis of bipolar depression. Psychiatr Serv Wash DC. 2001;52: 51–55. 10.1176/appi.ps.52.1.51 11141528

[43] Meyer F, Meyer TD. The misdiagnosis of bipolar disorder as a psychotic disorder: Some of its causes and their influence on therapy. J Affect Disord. 2009;112: 174–183. 10.1016/j.jad.2008.04.022 18555536

[44] Lasser K, Boyd J, Woolhandler S, Himmelstein D, McCormick D, Bor D. Smoking and mental illness: A population-based prevalence study. JAMA. 2000;284: 2606–2610. 10.1001/jama.284.20.2606 11086367

[45] Lawrence D, Mitrou F, Zubrick SR. Smoking and mental illness: results from population surveys in Australia and the United States. BMC Public Health. 2009;9: 285 10.1186/1471-2458-9-285 19664203 PMC2734850

[46] Ziedonis D, Hitsman B, Beckham JC, Zvolensky M, Adler LE, Audrain-McGovern J, et al Tobacco use and cessation in psychiatric disorders: National Institute of Mental Health report. Nicotine Tob Res Off J Soc Res Nicotine Tob. 2008;10: 1691–1715. 10.1080/14622200802443569 19023823

