# Supplementary material for: Smokers with Self-Reported Mental Health Conditions: A Case for Screening in the Context of Tobacco Cessation Services
Source: PLoS One. 2016 Jul 8;11(7):e0159127. doi: 10.1371/journal.pone.0159127 (PMC4938618; doi:10.1371/journal.pone.0159127)
Supplement: S1 Text — (DOCX) [file pone.0159127.s004.docx]

STROBE Statement—checklist of items that should be included in reports of observational studies

|  | Item No. | Recommendation | Page  No. | Relevant text from manuscript |
| --- | --- | --- | --- | --- |
| **Title and abstract** | 1 | (*a*) Indicate the study’s design with a commonly used term in the title or the abstract | 1 | Title |
|  |  | (*b*) Provide in the abstract an informative and balanced summary of what was done and what was found | 2 | Methods & Findings |
| Introduction | | | |  |
| Background/rationale | 2 | Explain the scientific background and rationale for the investigation being reported | 4 | Smoking prevalence is higher among those with mental health conditions (MHC) than among those without [1,2]. Smokers with MHC have a harder time quitting than smokers without MHC [3], and experience tobacco-related illnesses at a greater rate [4–8]. There has been a call for clinicians and researchers to focus more on tobacco cessation for people with MHC [9–14].  Tobacco cessation services, however, generally do not screen for MHC [12,15]. Screening for MHC can be time consuming, and many cessation service providers do not feel they are equipped to treat MHC [16–18]. On the other hand, mental health services tend to view tobacco dependence as a low priority compared with other treatment concerns [19–21]. The lack of screening for MHC in tobacco cessation services and low attention to tobacco dependence from mental health programs mean there are missed opportunities to help smokers with MHC in treatment settings [12].  This study presents a case for screening for MHC in tobacco cessation services, specifically in the context of a state quitline. Quitlines are telephone-based counseling services for smoking cessation [22]. They are major providers of tobacco cessation services in the U.S. The 51 state quitlines (including D.C.) collectively assist nearly 430,000 unique tobacco users annually [23]. There has been a growing interest in quitline utilization by smokers with MHC, and in what role quitlines can play in cessation efforts for these smokers [19,24–28]. |
| Objectives | 3 | State specific objectives, including any prespecified hypotheses | 5 | The main objective of this study is to show that a brief set of questions is useful in identifying smokers with self-reported MHC and in predicting outcomes for cessation. The questions do not aim to diagnose specific mental health conditions. Instead, they are used to generate information that could aid in treatment planning and counseling to improve cessation rates for smokers with MHC. |
| Methods | | | |  |
| Study design | 4 | Present key elements of study design early in the paper | 5 | To test this approach, we embedded a self-report measure for mental health in the intake protocol of the California Smokers’ Helpline, the longest running state quitline in the U.S. The present study examines the feasibility and usefulness of employing such a measure. Specifically, the value of the measure was tested by examining if answers to the MHC questions predicted treatment utilization and smoking cessation outcomes. |
| Setting | 5 | Describe the setting, locations, and relevant dates, including periods of recruitment, exposure, follow-up, and data collection | 6-8 | Study participants were adult smokers who called the California Smokers’ Helpline (CSH) between June 2012-September 2015. CSH is California’s quitline, a free statewide telephone-based tobacco cessation service. The Helpline provides services in English, Spanish, Mandarin, Cantonese, Vietnamese, and Korean. This study included only those who called the English and Spanish language lines. Since participants lived in different locations throughout California, and took part in the study by telephone, oral consent was obtained and recorded in the Helpline data base. All research activity for this study, including the consent procedure, was approved by the University of California, San Diego Human Research Protections Program (#080517).  **Procedure**  *Intake*  Helpline staff used a computer-assisted telephone interview (CATI) system that presented the mental health questions embedded in the Helpline standard intake. The standard intake interview included items such as demographics, health insurance status, smoking status, tobacco consumption, and health conditions. The mental health questions followed questions on physical health.  *Services*  All counselors’ contact with participants was logged in the Helpline database, including call date, call attempts, and type and length of calls. The counseling protocol used during this study has been described elsewhere in detail [30] and has been shown to be effective in clinical trials [31,32]. Counseling was provided by trained counselors, and included a comprehensive initial session to prepare for a quit date and up to four follow-up calls at critical times, based on the probability of relapse [33].  *Measures*  Baseline measures to assess MHC at intake were consistent with those recommended by the North American Quitline Consortium (NAQC) as an optional addition to the minimal data set [34]. Helpline callers were asked “Do you have any mental health conditions such as an anxiety disorder? Depression disorder? Bipolar disorder? Schizophrenia? Alcohol/drug abuse?” Staff recorded a yes or no for each condition. When smokers stated they did not know (about 1% of the time), staff followed with, “Has a doctor or other health care professional told you that you have a mental health condition?” If smokers responded yes, they were asked to specify which conditions, and staff coded responses accordingly.  The evaluation measure used to determine engagement in services was number of counseling sessions received. Quitting was assessed by two measures: quit attempts and prolonged abstinence. A quit attempt was defined as quitting for at least 24 hours. For those that made a quit attempt, prolonged abstinence was defined as not smoking for at least 180 days at the time of evaluation. If a smoker made more than one quit attempt, the first quit attempt was used in the analysis of prolonged abstinence.  *Evaluation*  Seven-month post enrollment evaluation interviews were conducted by telephone by research assistants, independent of the counselors. Based on standard Helpline evaluation procedures, with oversampling of pregnant smokers and households with children under age five, an average of 9% of participants were randomly selected for evaluation. Evaluations were conducted from January 2013-February 2015. |
| Participants | 6 | (*a*) *Cohort study*—Give the eligibility criteria, and the sources and methods of selection of participants. Describe methods of follow-up  *Case-control study*—Give the eligibility criteria, and the sources and methods of case ascertainment and control selection. Give the rationale for the choice of cases and controls  *Cross-sectional study*—Give the eligibility criteria, and the sources and methods of selection of participants | 6-8 | Study participants were adult smokers who called the California Smokers’ Helpline (CSH) between June 2012-September 2015. CSH is California’s quitline, a free statewide telephone-based tobacco cessation service. The Helpline provides services in English, Spanish, Mandarin, Cantonese, Vietnamese, and Korean. This study included only those who called the English and Spanish language lines. Since participants lived in different locations throughout California, and took part in the study by telephone, oral consent was obtained and recorded in the Helpline data base. All research activity for this study, including the consent procedure, was approved by the University of California, San Diego Human Research Protections Program (#080517).  **Procedure**  *Intake*  Helpline staff used a computer-assisted telephone interview (CATI) system that presented the mental health questions embedded in the Helpline standard intake. The standard intake interview included items such as demographics, health insurance status, smoking status, tobacco consumption, and health conditions. The mental health questions followed questions on physical health.  *Measures*  Baseline measures to assess MHC at intake were consistent with those recommended by the North American Quitline Consortium (NAQC) as an optional addition to the minimal data set [34]. Helpline callers were asked “Do you have any mental health conditions such as an anxiety disorder? Depression disorder? Bipolar disorder? Schizophrenia? Alcohol/drug abuse?” Staff recorded a yes or no for each condition. When smokers stated they did not know (about 1% of the time), staff followed with, “Has a doctor or other health care professional told you that you have a mental health condition?” If smokers responded yes, they were asked to specify which conditions, and staff coded responses accordingly.  The evaluation measure used to determine engagement in services was number of counseling sessions received. Quitting was assessed by two measures: quit attempts and prolonged abstinence. A quit attempt was defined as quitting for at least 24 hours. For those that made a quit attempt, prolonged abstinence was defined as not smoking for at least 180 days at the time of evaluation. If a smoker made more than one quit attempt, the first quit attempt was used in the analysis of prolonged abstinence. |
|  |  | (*b*) *Cohort study*—For matched studies, give matching criteria and number of exposed and unexposed  *Case-control study*—For matched studies, give matching criteria and the number of controls per case | NA |  |
| Variables | 7 | Clearly define all outcomes, exposures, predictors, potential confounders, and effect modifiers. Give diagnostic criteria, if applicable | 7-8 | *Measures*  Baseline measures to assess MHC at intake were consistent with those recommended by the North American Quitline Consortium (NAQC) as an optional addition to the minimal data set [34]. Helpline callers were asked “Do you have any mental health conditions such as an anxiety disorder? Depression disorder? Bipolar disorder? Schizophrenia? Alcohol/drug abuse?” Staff recorded a yes or no for each condition. When smokers stated they did not know (about 1% of the time), staff followed with, “Has a doctor or other health care professional told you that you have a mental health condition?” If smokers responded yes, they were asked to specify which conditions, and staff coded responses accordingly.  The evaluation measure used to determine engagement in services was number of counseling sessions received. Quitting was assessed by two measures: quit attempts and prolonged abstinence. A quit attempt was defined as quitting for at least 24 hours. For those that made a quit attempt, prolonged abstinence was defined as not smoking for at least 180 days at the time of evaluation. If a smoker made more than one quit attempt, the first quit attempt was used in the analysis of prolonged abstinence. |
| Data sources/ measurement | 8* | For each variable of interest, give sources of data and details of methods of assessment (measurement). Describe comparability of assessment methods if there is more than one group | 6-8 | Study participants were adult smokers who called the California Smokers’ Helpline (CSH) between June 2012-September 2015. CSH is California’s quitline, a free statewide telephone-based tobacco cessation service. The Helpline provides services in English, Spanish, Mandarin, Cantonese, Vietnamese, and Korean. This study included only those who called the English and Spanish language lines. Since participants lived in different locations throughout California, and took part in the study by telephone, oral consent was obtained and recorded in the Helpline data base. All research activity for this study, including the consent procedure, was approved by the University of California, San Diego Human Research Protections Program (#080517).  **Procedure**  *Intake*  Helpline staff used a computer-assisted telephone interview (CATI) system that presented the mental health questions embedded in the Helpline standard intake. The standard intake interview included items such as demographics, health insurance status, smoking status, tobacco consumption, and health conditions. The mental health questions followed questions on physical health.  *Services*  All counselors’ contact with participants was logged in the Helpline database, including call date, call attempts, and type and length of calls. The counseling protocol used during this study has been described elsewhere in detail [30] and has been shown to be effective in clinical trials [31,32]. Counseling was provided by trained counselors, and included a comprehensive initial session to prepare for a quit date and up to four follow-up calls at critical times, based on the probability of relapse [33].  *Measures*  Baseline measures to assess MHC at intake were consistent with those recommended by the North American Quitline Consortium (NAQC) as an optional addition to the minimal data set [34]. Helpline callers were asked “Do you have any mental health conditions such as an anxiety disorder? Depression disorder? Bipolar disorder? Schizophrenia? Alcohol/drug abuse?” Staff recorded a yes or no for each condition. When smokers stated they did not know (about 1% of the time), staff followed with, “Has a doctor or other health care professional told you that you have a mental health condition?” If smokers responded yes, they were asked to specify which conditions, and staff coded responses accordingly.  The evaluation measure used to determine engagement in services was number of counseling sessions received. Quitting was assessed by two measures: quit attempts and prolonged abstinence. A quit attempt was defined as quitting for at least 24 hours. For those that made a quit attempt, prolonged abstinence was defined as not smoking for at least 180 days at the time of evaluation. If a smoker made more than one quit attempt, the first quit attempt was used in the analysis of prolonged abstinence.  *Evaluation*  Seven-month post enrollment evaluation interviews were conducted by telephone by research assistants, independent of the counselors. Based on standard Helpline evaluation procedures, with oversampling of pregnant smokers and households with children under age five, an average of 9% of participants were randomly selected for evaluation. Evaluations were conducted from January 2013-February 2015. |
| Bias | 9 | Describe any efforts to address potential sources of bias | N/A |  |
| Study size | 10 | Explain how the study size was arrived at | 6,8 | Study participants were adult smokers who called the California Smokers’ Helpline (CSH) between June 2012-September 2015. CSH is California’s quitline, a free statewide telephone-based tobacco cessation service. The Helpline provides services in English, Spanish, Mandarin, Cantonese, Vietnamese, and Korean. This study included only those who called the English and Spanish language lines. Since participants lived in different locations throughout California, and took part in the study by telephone, oral consent was obtained and recorded in the Helpline data base. All research activity for this study, including the consent procedure, was approved by the University of California, San Diego Human Research Protections Program (#080517).  **Procedure**  *Intake*. Helpline staff used a computer-assisted telephone interview (CATI) system that presented the mental health questions embedded in the Helpline standard intake. The standard intake interview included items such as demographics, health insurance status, smoking status, tobacco consumption, and health conditions. The mental health questions followed questions on physical health.  A total of 125,261 adult smokers who called the quitline were asked the mental health questions during the study period. |

Continued on next page

| Quantitative variables | 11 | Explain how quantitative variables were handled in the analyses. If applicable, describe which groupings were chosen and why | N/A |  |
| --- | --- | --- | --- | --- |
| Statistical methods | 12 | (*a*) Describe all statistical methods, including those used to control for confounding | 8 | *Statistical analysis*.  To examine the effect of number of MHC on treatment engagement, all participants were categorized into three groups: those with no MHC, one MHC, and more than one MHC. Confidence intervals (95%) were calculated around parameters and chi-squares were used to evaluate differences in proportions. Survival analysis was performed by using Kaplan-Meier method to generate relapse curves for the three MHC groups. Log-rank tests with Sidak multiple-comparison adjustment were used to compare the relapse curves among the three MHC groups. All statistical analyses were conducted using SAS statistical package, version 9.4 [35]. |
|  |  | (*b*) Describe any methods used to examine subgroups and interactions | N/A |  |
|  |  | (*c*) Explain how missing data were addressed | N/A |  |
|  |  | (*d*) *Cohort study*—If applicable, explain how loss to follow-up was addressed  *Case-control study*—If applicable, explain how matching of cases and controls was addressed  *Cross-sectional study*—If applicable, describe analytical methods taking account of sampling strategy | 8 | *Statistical analysis*. To examine the effect of number of MHC on treatment engagement, all participants were categorized into three groups: those with no MHC, one MHC, and more than one MHC. Confidence intervals (95%) were calculated around parameters and chi-squares were used to evaluate differences in proportions. Quit attempt rates and 180-day abstinence rates were also obtained from participants reached for evaluation. Log-rank tests were used to compare the relapse curves. All statistical analyses were conducted using SAS statistical package, version 9.4 [34]. |
|  |  | (*e*) Describe any sensitivity analyses | N/A |  |
| Results | | | | |
| Participants | 13* | (a) Report numbers of individuals at each stage of study—eg numbers potentially eligible, examined for eligibility, confirmed eligible, included in the study, completing follow-up, and analysed | 8 | A total of 125,261 adult smokers who called the quitline were asked the mental health questions during the study period. |
|  |  | (b) Give reasons for non-participation at each stage | N/A |  |
|  |  | (c) Consider use of a flow diagram | N/A |  |
| Descriptive data | 14* | (a) Give characteristics of study participants (eg demographic, clinical, social) and information on exposures and potential confounders | 9 | Table 1 |
|  |  | (b) Indicate number of participants with missing data for each variable of interest | 11-12 | Table 2 and 3 |
|  |  | (c) *Cohort study*—Summarise follow-up time (eg, average and total amount) | N/A |  |
| Outcome data | 15* | *Cohort study*—Report numbers of outcome events or summary measures over time | N/A |  |
|  |  | *Case-control study—*Report numbers in each exposure category, or summary measures of exposure | N/A |  |
|  |  | *Cross-sectional study—*Report numbers of outcome events or summary measures | 9,11,12 | Tables 1, 2 and 3 |
| Main results | 16 | (*a*) Give unadjusted estimates and, if applicable, confounder-adjusted estimates and their precision (eg, 95% confidence interval). Make clear which confounders were adjusted for and why they were included | 9,11,12 | Tables 1, 2 and 3 |
|  |  | (*b*) Report category boundaries when continuous variables were categorized | 8 | To examine the effect of number of MHC on treatment engagement, all participants were categorized into three groups: those with no MHC, one MHC, and more than one MHC. |
|  |  | (*c*) If relevant, consider translating estimates of relative risk into absolute risk for a meaningful time period | N/A |  |

Continued on next page

| Other analyses | 17 | Report other analyses done—eg analyses of subgroups and interactions, and sensitivity analyses | N/A |  |
| --- | --- | --- | --- | --- |
| Discussion | | | | |
| Key results | 18 | Summarise key results with reference to study objectives | 13-17 | Results from this study of over 125,000 smokers showed that a simple self-report measure of MHC can be used as part of a routine quitline intake. This measure was informative, as item responses were associated with treatment utilization and smoking cessation rates. Three results stand out: First, a large proportion of smokers calling the statewide service reported at least one mental health condition (MHC). Second, those who reported MHC tended to use more counseling. Third, callers with or without MHC were all highly motivated to make a quit attempt, but those with MHC were more likely to relapse after a quit attempt. |
| Limitations | 19 | Discuss limitations of the study, taking into account sources of potential bias or imprecision. Discuss both direction and magnitude of any potential bias | 17 | The main limitation of this study is the use of non-validated self-report questions to assess MHC. Brief screening questions for conditions such as depression and anxiety can result in false positives [36]. Smokers may have responded as if asked whether they *ever* had MHC, rather than *currently* had MHC as implied in the question. “*Do you have* any mental health conditions such as…” The intake protocol in this study did not require smokers to clarify when they received their MHC diagnoses, which could lead to higher than usual endorsement of multiple conditions. |
| Interpretation | 20 | Give a cautious overall interpretation of results considering objectives, limitations, multiplicity of analyses, results from similar studies, and other relevant evidence | 17-18 | Smokers with MHC call quitlines in large numbers. They are motivated to quit, but less likely to succeed in quitting than smokers with no MHC. Thus, it is imperative to develop interventions to help them quit successfully. Telephone quitlines are one of the few behavioral services that reach smokers in large numbers and these programs have been disseminated internationally [22]. However, the efficacy of these quitlines for smokers with MHC is not well established. This is in part due to the fact that most cessation services do not measure MHC or do not use it to guide the treatment. This study shows that even a brief measure of MHC can predict the relapse probability of a given quit attempt. This suggests that smoking cessation services should routinely assess for MHC and use the information to guide treatment. One way to improve quit rates for smokers with MHC is to employ protocols that have more counseling sessions or longer use of cessation pharmacotherapy [10,16,46]. Another way, especially for those with more than one MHC, would be more intensive interventions along with referral to and collaboration with mental health clinicians for concurrent mental health treatment. A more formal test of whether a more intensive treatment can produce a higher quit rate for smokers with MHC is needed.  As a starting point, quitlines could use a general measure of psychiatric health at intake to identify smokers with MHC, such as the one used in this study. The fact that a high number of smokers with MHC call quitlines for assistance presents an excellent opportunity to increase tobacco dependency treatment utilization in people with MHC, thereby improving their quality of life. Quitline services are free, easy to access, and provided by well-trained staff. They can continue to play a role in helping smokers with MHC quit, and can be a resource for mental health clinicians working with smokers with MHC. |
| Generalisability | 21 | Discuss the generalisability (external validity) of the study results | 17-18 |  |
| Other information | |  | | |
| Funding | 22 | Give the source of funding and the role of the funders for the present study and, if applicable, for the original study on which the present article is based | Metadata | Journal requested that funding not be mentioned in the manuscript. |

*Give information separately for cases and controls in case-control studies and, if applicable, for exposed and unexposed groups in cohort and cross-sectional studies.

**Note:** An Explanation and Elaboration article discusses each checklist item and gives methodological background and published examples of transparent reporting. The STROBE checklist is best used in conjunction with this article (freely available on the Web sites of PLoS Medicine at http://www.plosmedicine.org/, Annals of Internal Medicine at http://www.annals.org/, and Epidemiology at http://www.epidem.com/). Information on the STROBE Initiative is available at www.strobe-statement.org.
