# Supplementary material for: Smokers with Self-Reported Mental Health Conditions: A Case for Screening in the Context of Tobacco Cessation Services
Source: PLoS One. 2016 Jul 8;11(7):e0159127. doi: 10.1371/journal.pone.0159127 (PMC4938618; doi:10.1371/journal.pone.0159127)
Supplement: S2 Text — (DOCX) [file pone.0159127.s005.docx]

**MH-t1t2.csv** (data for the results in Table 1 and Table 2)

anxi: Anxiety disorder (1=yes, 0=no)

depr: Depression disorder (1=yes, 0=no)

bipo: Bipolar disorder (1=yes, 0=no)

schi: Schizophrenia (1=yes, 0=no)

drug_alcoh: Alcohol/drug abuse (1=yes, 0=no)

any_mhc: Had any one of the MHC (1=yes, 0=no)

n_mhc: Number of Mental Health Condition

mhc: Mental Health Condition groups (0=0 MHC, 1=1 MHC, 2=more than 1 MHC)

age: 2_18-24, 3_25-44, 4_45-64, 5_65+

gender: 1_M=Male, 2_F=Female

edu: 1= GED/Diploma or high school or lower, 2=At least some college

race: 1_White, 2_Black, 3_Hispanic, 4_Asian, 5_Al, 6_Multi, 7_Other, 8_Don’t know)

**MH-t3f1.csv** (data for the results in Table 3 and Figure 1)

mhc: Mental Health Condition groups (0=0 MHC, 1=1 MHC, 2=more than 1 MHC)

ic: received initial session (1=yes, 0=no)

ts_3: total sessions received >=3 (1=yes, 0=no)

med: Use of any pharmacotherapy (1=yes, 0=no)

attempt: Quit attempt (1=yes, 0=no)

**MH-f2.csv** (data for the results in Figure2)

mhc: Mental Health Condition groups (0=0 MHC, 1=1 MHC, 2=more than 1 MHC)

time_180: Days after quitting smoking

censor_180: Censor (0=smoking, 1=not smoking, censored)
